# Cervical epidural hematoma post-trauma: a case report

**DOI:** 10.11604/pamj.2022.43.12.28877

**Published:** 2022-09-07

**Authors:** Abderrahmane Housni, Nourou Dine Adeniran Bankole, Abdessamad El Ouahabi

**Affiliations:** 1 Service de Neurochirurgie de l’Hôpital Des Spécialités, Université Mohammed V de Rabat, Rabat, Maroc

**Keywords:** Spinal epidural hematoma, cervical spine, trauma, cord compression, case report

## Abstract

Post-traumatic spinal epidural cervical hematoma is defined as a collection of blood at the level of the epidural space following a trauma. It remains a rare presentation. We report here the case of a cervical epidural hematoma extending from C3 to C5, in a 55-year-old patient victim of a public traffic accident admitted one hour after trauma. Computed Tomography (CT) scan found a compressive epidural hematoma extending C3 to C5; the patient underwent a posterior surgical approach, which allowed to evacuate the hematoma. This rare clinical entity is an emergency diagnosis and management, which needs collaboration between, Intensive Care Unit (ICU) specialists, neurosurgeons, neuroradiologists, and physiotherapists for good outcomes and follow-up.

## Introduction

Spinal epidural hematoma (SHE) is a rare entity, it often occurs in the context of taking anticoagulants or else in elderly subjects with comorbidities. The traumatic context remains extremely rare; it could be found in isolated cervical trauma or the context of a polytrauma. An incidence of 2.5% was reported for post-traumatic cervical spine epidural hematoma [1]. We present a case of spinal cervical epidural hematoma after trauma in an adult patient.

## Patient and observation

### Case description

**Clinical finding:** a 55-year-old male patient, with no specific history, suffered head trauma following a road accident, initially, the patient lost consciousness with a spontaneous return to consciousness. On admission the patient was conscious, Glasgow Coma Scale (GCS) 14 agitated with breathing disorders, pupil equal and reactive, hemodynamically stable, with no apparent sensory-motor deficit. The patient was immediately admitted to Intensive Care Unit (ICU) and intubated with sedation. We were not able to identify before surgical management if he had a neurologic deficit or not because he presented with breathing disorders and was intubated.

**Figure 1:**
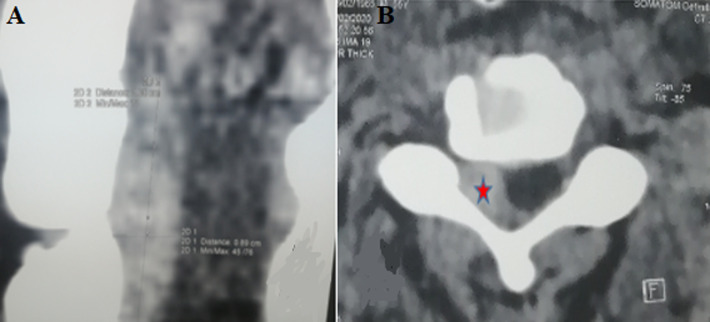
A) CT scan sagittal showed cervical epidural hematoma, extending from C3 to C5; B) CT scan axial view showing hematoma (red stars) compressing the spinal cord on the right side without associated bone lesion

**Diagnosis assessment:** our patient benefited from CT Body, which showed intercostal fractures with a cervical epidural hematoma, extending from C3 to C5 compressing the spinal cord on the right side without associated bone lesion (Figure 1). Moreover, brain imaging showed cerebral edema without hematoma or skull fracture lesions. The biological exam returned normal without particularities of hemostasis disorders.

**Management:** the patient was admitted urgently to the operating room, he benefited from a posterior cervical approach laminotomy, intraoperatively we discovered an important cervical epidural hematoma extending from C3 to C5 driving back the spinal cord on the left side with square roots, which dilated. The hematoma was fresh and its evacuation was laborious we proceeded to the achievement of good hemostasis. After surgery patient was admitted to ICU and stayed 10 days for further management. At discharge, the patient had a focal motor neurological deficit in the right upper extremity especially the c5 and c6 territory evaluated about 3/5. We send him to rehabilitation for physiotherapy management.

**Figure 2:**
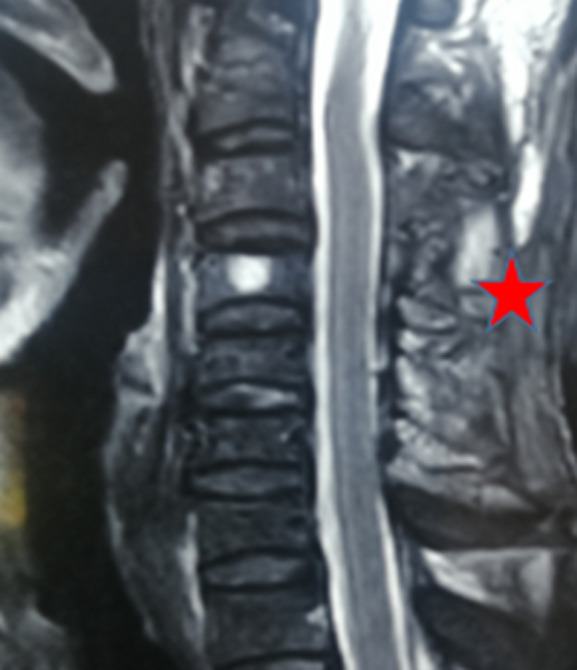
magnetic resonance imaging sagittal view postoperative follow-up showing C3-C5 laminectomy (red stars) without hematoma

**Follow-up:** during a follow-up of 6 months, after discharge, the neurological function of the patient did not greatly recover. The motor deficit remains at 4/5. A cervical Magnetic Resonance Imaging (MRI) realized 6 months after the surgery showed that the hematoma has been completely removed (Figure 2).

**Informed consent:** the patient gives informed consent.

## Discussion

In 1682 Jackson R *et al*. described for the first time Spinal hematoma after autopsies and as a clinical diagnosis since 1867 [2]. At this time, 300 cases were reported in the literature [3]. Spinal epidural hematomas (SEH) is idiopathic without finding the origin of the bleeding, and spontaneous when it results from a minor trauma [3]. The incidence of SEH is about 0.1 per 100.000 per year, men are slightly more affected than women [4] and account for less than 1% of all spinal canal space-occupying lesions [5]. Traumatic SEHs occur frequently in adults over 40 years old, mostly in the cervical and proximal thoracic spine [3]. Our patient was a 55-year-old-man. The origin of the bleeding is most often venous, linked to the richness of the epidural venous network made of veins poor in valves and vulnerable, with even minimal trauma sometimes. The arterial origin might be evoked in front of the rapidity of the installation of clinical signs, the frequent association with arterial hypertension, and the lateral localization of the hematoma has been described [6]. However, the etiopathogenesis of the SEH is still unclear.

Anticoagulant therapy or blood coagulopathies constitute a most factors risk, neoplasms, or degenerative spinal diseases, increases risk of SHE [6,7]. There are also certain descriptions of iatrogenic hematoma, namely practice acupuncture, spinal needling, post-discectomy hematomas [8,9], or some traumatic massage or manipulation [10]. However, some idiopathic cases of spontaneous hematoma have been described [11]. Our case occurred after trauma, the hematoma sits mainly in the right lateral, compressing the roots of the same side and driving back the cervical spinal cord at this level on the contralateral side. The spinal epidural hematoma has several clinical manifestations, which makes its diagnosis quite difficult, it manifests itself most often by simple neck pain, even a partial or total deficit depending on the size and importance of the hematoma, and often underdiagnosed, especially if it comes in the context of polytrauma [6]. In our case, it was a context of polytrauma, a state of consciousness that did not allow a neurological deficit noted; especially the attention much more focused on a probable surgical brain injury. We, therefore, recall that any isolated head trauma is, until proof to the contrary, a cervical spine trauma.

The CT-scan makes it possible to have the diagnosis initially by showing hyperdense biconvex imaging in the level and especially the associated bone lesions. Magnetic resonance imaging remains the preferred examination allowing a good visualization of the hematoma, which appears in T2 hyperintense, its extent, its effect on the spinal cord and adjacent structures [7]. Magnetic resonance imaging also provides very important information about soft parts such as muscles, ligaments, it helps us to better understand the mechanism of the bleeding and to choose the best treatment [3]. Unfortunately, this exam is not available in the emergency department of some hospitals [10]. In our case, we use only CT-scan to assess the diagnosis, we do not have MRI in our Emergency Department.

Regarding treatment, our review of the literature has shown that the two attitudes are the same, namely a surgical approach [11-13] or conservative treatment [9,14,15], associated in all cases with boluses of corticosteroids with analgesic treatment. Staying in ICU depends on the general clinical state of the patient at admission. Rehabilitation remains an important place in overall care by insisting on the fact that the establishment must be early and which guarantees better results.

## Conclusion

Post-traumatic spinal epidural cervical hematoma remains rare. We should always think about that for each isolated head trauma or polytrauma following an accident on the public road even outside clinical manifestation by systematically performing imaging. The earliness of adequate care strongly conditions the prognosis and the correct involvement of all stakeholders such as paramedics, emergency physicians, resuscitators, neurosurgeons, rehabilitators are necessary to obtain better outcomes.
